# Wedge hybrid plasmonic THz waveguide with long propagation length and ultra-small deep-subwavelength mode area

**DOI:** 10.1038/srep11457

**Published:** 2015-07-09

**Authors:** Chengcheng Gui, Jian Wang

**Affiliations:** 1 Wuhan National Laboratory for Optoelectronics, School of Optical and Electronic Information, Huazhong University of Science and Technology, Wuhan 430074, Hubei, China

## Abstract

We present a novel design of wedge hybrid plasmonic terahertz (THz) waveguide consisting of a silicon (Si) nanowire cylinder above a triangular gold wedge with surrounded high-density polyethylene as cladding. It features long propagation length and ultra-small deep-subwavelength mode confinement. The mode properties of wedge hybrid plasmonic THz waveguide are comprehensively characterized in terms of propagation length (*L*), normalized mode area (*Aeff* /*A*_*0*_), figure of merit (*FoM*), and chromatic dispersion (*D*). The designed wedge hybrid plasmonic THz waveguide enables an ultra-small deep-subwavelength mode area which is more than one-order of magnitude smaller compared to previous rectangular one. When choosing the diameter of Si nanowire cylinder, a smaller diameter (e.g. 10 μm) is preferred to achieve longer *L* and higher *FoM*, while a larger diameter (e.g. 60 μm) is favorable to obtain smaller *Aeff* /*A*_*0*_ and higher *FoM*. We further study the impacts of possible practical fabrication errors on the mode properties. The simulated results of propagation length and normalized mode area show that the proposed wedge hybrid plasmonic THz waveguide is tolerant to practical fabrication errors in geometry parameters such as misalignment in the horizontal direction, variation of wedge tip angle, and variation of wedge tip curvature radius.

Terahertz (THz) radiation, also called terahertz waves or T-waves with occupied electromagnetic spectra between microwaves and infrared light waves, has received considerable attention for important applications in a wide range of fields, such as sensing, imaging, and spectroscopy^1^,^2^,^3^. Among various scientific research areas of THz waves, guided THz wave propagation is a challenge one. Efficient transmission of THz signals has driven the great demand for compact, low-loss, and well-confined THz guiding structures which could enable high-density integration of THz functionalities and facilitate high-speed long-range ultra wide band THz communications. In the recent years, THz waveguides have attracted increasing interest for offering efficient and flexible low-loss interconnects and communications channels. Different types of THz waveguides have been reported. For instance, dielectric-based THz waveguides such as polymer^4^ and plastic ribbon planar waveguides^5^ show large mode area together with low propagation loss which is due to residual material absorption (power absorption coefficient α ~ 1 cm^−1^). In addition to dielectric-based THz waveguides, metal is also considered to be an alternative and promising candidate for THz guiding. Generally speaking, surface plasmon polaritons (SPPs) are always involved in metal-based devices. SPPs are known as surface electromagnetic waves which are well confined to metal-dielectric interfaces. However, it is worth noting that metal almost resembles a perfect electric conductor at THz frequency since its plasma frequency is always in the ultraviolet part of the electromagnetic spectrum, leading to SPPs highly delocalized on metal surfaces^6^,^7^. A possible solution is proposed and the existing surface plasmons frequency is achievable by cutting holes or grooves in metal surfaces to increase the penetration of surface electromagnetic fields into the metal^8^,^9^,^10^. Remarkably, metal-based THz waveguides tend to exhibit lower propagation loss compared to their dielectric counterparts owing to the larger conductivities of metal at THz frequencies. Despite these features, for metallic structures, there is also a fundamental trade-off between strong mode confinement and low attenuation characteristics. For example, cylindrical metal wire (α ~ 0.02 cm^−1^)^11^ allows for low attenuation of SPPs but cannot support well-confined surface waves. Metallic slit waveguide^12^ offers well-confined mode but suffers from relatively high attenuation. Different kinds of air- or dielectric-filled metallic parallel plate waveguides are employed to support THz transmission with millimeter-scale propagation length and relatively large mode area^13^,^14^,^15^.

Generally speaking, mode confinement and propagation loss are intrinsically correlated. A hybrid plasmonic waveguide is proposed to improve the trade-off between the mode confinement and propagation loss at telecommunications frequency^16^. The hybrid plasmonic waveguide formed by placing a cylindrical dielectric nanowire on a metal surface can offer tight mode confinement with moderate loss. Then, several structures of hybrid plasmonic waveguides have been proposed and studied^17^,^18^,^19^,^20^,^21^. The interaction between dielectric waveguide mode and pure SPP mode can achieve longer propagation length while maintaining relatively small mode size. Very recently, a rectangular hybrid THz waveguide has been proposed showing subwavelength mode confinement and reasonably low loss^22^. The working principle of such structure is similar to the one working at the telecommunications frequency^16^. For the rectangular hybrid THz waveguide, further shrinking of the mode area seems to be difficult due to limited reduction of the low-index gap region. It is noted that at telecommunications frequency one may use wedge waveguide structure to further reduce the mode area^23^,^24^. In this scenario, a laudable goal would be to explore wedge hybrid waveguide operating at THz frequency to push more the tight mode confinement.

In this paper, we present a new approach of THz guiding using fundamental hybrid plasmonic mode which is coupled by the wedge plasmonic mode and dielectric mode. The proposed wedge hybrid plasmonic THz waveguide features long propagation length and ultra-small deep-subwavelength mode confinement. We further investigate the impacts of possible practical fabrication errors on the mode properties.

## Results

### Waveguide structure and mode distributions

**Figure 1:**
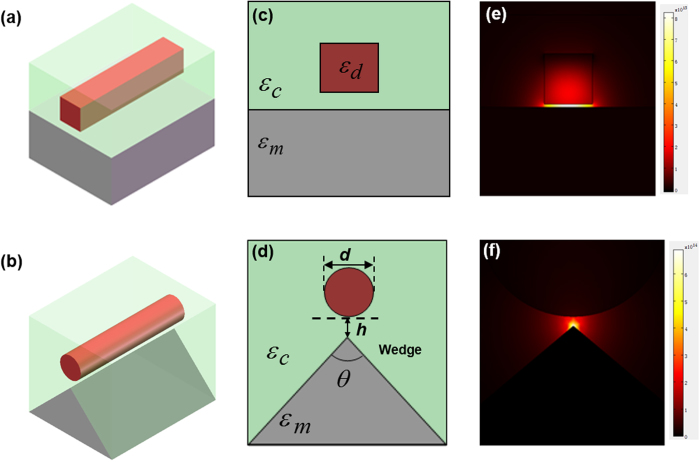
(**a**)(**b**) 3D layout waveguide structures, (**c**)(**d**) cross-section structures, and (**e**)(**f**) mode distributions of (a)(c)(**e**) rectangular hybrid plasmonic THz waveguide and (b)(d)(f) wedge hybrid plasmonic THz waveguide.

Shown in Fig. 1(a) are 3D layout of a rectangular hybrid THz waveguide and the proposed wedge hybrid plasmonic THz waveguide. Figure 1(c)–(f) depict corresponding cross-section structures and mode distributions of rectangular and wedge hybrid plasmonic THz waveguides. In the designed wedge hybrid plasmonic THz waveguide, a float-zone high-resistivity silicon (Si) nanowire cylinder, which has a diameter of *d* and a permittivity of *ε*_*d*_ = 11.6827 at 1  THz^25^, is placed above a triangular gold wedge as the metal substrate. The permittivity of gold is obtained by the Drude model, *ε*(*ω*)=1-*ω*^2^_*p*_/[*ω*(*ω+iω*_c_)], where *ω*_*p*_ = 1.37 × 10^16^ Hz and *ω*_c_ = 4.07 × 10^13^Hz^13^. The cladding is high-density polyethylene (HDPE) with a permittivity of *ε*_*c*_ =2.37 at 1 THz^26^. The gap height between the Si nanowire cylinder and the tip of wedge is h and the tip angle is (0 < θ < 180) degree (deg). Mode properties are studied using the finite-element method (FEM) software package COMSOL Multiphysics with the scattering bound condition and rectangular perfectly matched layer (PML). Compared to the mode distribution of rectangular hybrid plasmonic THz waveguide as shown in Fig. 1(e), it can be seen from Fig. 1(f) that wedge hybrid plasmonic THz waveguide features greatly reduced deep-subwavelength mode confinement.

### Mode properties

**Figure 2:**
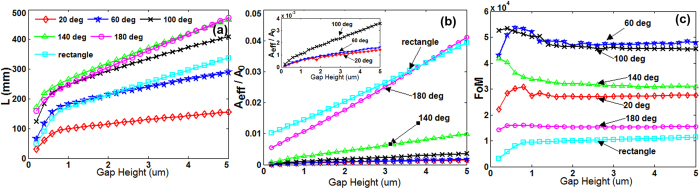
(**a**) Propagation length (*L*), (**b**) normalized mode area (*A*_*eff*_ */A*_*0*_), and (**c**) figure of merit (*FoM*) vs. gap height (*h*) under different wedge tip angles of the wedge hybrid plasmonic THz waveguide. The diameter of Si nanowire cylinder is 40 μm. The rectangular hybrid THz waveguide is also plotted for comparison (rectangle).

We further characterize the mode properties of wedge hybrid plasmonic THz waveguide. Propagation length (*L*), normalized mode area (*A*_*eff*_ */A*_*0*_), and figure of merit (*FoM*) of the hybrid mode of wedge THz waveguide are shown in Fig. 2. The gap height (*h*) changes from 0.2 to 5 μm. The diameter of Si nanowire cylinder is 40 μm. The tip angle is set at 20, 60, 100, 140 and 180 deg, respectively.

As shown in Fig. 2, *L* and *A*_*eff*_ */A*_*0*_ increase while *FoM* keeps almost unchanged with the increase of the gap height under different tip angles. For comparison, the mode properties of rectangular hybrid plasmonic THz waveguide are also shown. Some more conclusions can be deduced from Fig. 2 as follows. 1) For the wedge hybrid plasmonic THz waveguide, the *L* and *A*_*eff*_ */A*_*0*_ decrease when reducing the tip angle. 2) The wedge hybrid plasmonic THz waveguide features higher *FoM* than the rectangular one under different tip angles. With the increase of tip angle, the *FoM* increases first and then decreases. 3) Even under 180 deg of the tip angle, similar *A*_*eff*_ */A*_*0*_, longer L and higher *FoM* are achievable compared to rectangular one, which is due to the Si nanowire cylinder adopted in the structure. By comprehensively considering *L, A*_*eff*_ */A*_*0*_ and *FoM* as shown in Fig. 2(a–c), one would prefer to choose an optimized tip angle of 100 deg. Under such optimal tip angle of 100 deg and a gap height of 1 μm, the obtained *L, A*_*eff*_ */A*_*0*_ and *FoM* of the designed wedge hybrid plasmonic THz waveguide improve 1.4, 13.5 and 5.3 times compared to the rectangular one. In particular, the designed wedge hybrid plasmonic THz waveguide offers an ultra-small deep-subwavelength mode area which is more than one-order of magnitude smaller compared to the rectangular one.

**Figure 3:**
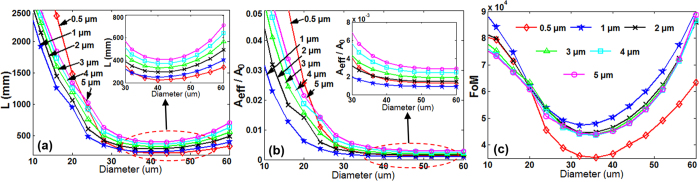
(**a**) Propagation length (*L*), (**b**) normalized mode area (*A*_*eff*_ */A*_*0*_), and (**c**) figure of merit (*FoM*) vs. diameter of Si nanowire cylinder (***d***) under different gap heights of the wedge hybrid THz waveguide. The wedge tip angle is 100 deg.

Figure 3 shows propagation length (*L*), normalized mode area (*A*_*eff*_ */A*_*0*_) and figure of merit (*FoM*) of wedge hybrid plasmonic THz waveguide as a function of the diameter of Si nanowire cylinder under optimized tip angle of 100 deg and different gap height of 0.5, 1, 2, 3, 4, 5 μm, respectively. With the increase of diameter of Si nanowire cylinder, the *L* decreases first and then increases slightly while the *A*_*eff*_ */A*_*0*_ decreases. Thus the *FoM* decreases first and then increases. According to different requirements, one may choose different values of diameter. That is, a smaller diameter (e.g. 10 μm) is preferred when expecting longer *L* and higher *FoM*, while a larger diameter (e.g. 60 μm) is favorable when requiring smaller *A*_*eff*_ */A*_*0*_ and higher *FoM*.

**Figure 4:**
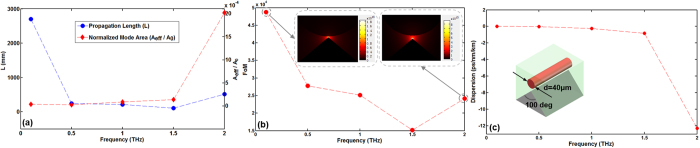
(**a**) Propagation length (*L*) and normalized mode area (*A*_*eff*_ */A*_*0*_), (**b**) figure of merit (*FoM*), and (**c**) chromatic dispersion vs. frequency from 0.1 to 2 THz. The wedge tip angle is 100 deg, the diameter of Si nanowire cylinder is 40 μm, and the gap height is 0.2 μm.

We also evaluate the propagation length (*L*), normalized mode area (*A*_*eff*_ */A*_*0*_), figure of merit (*FoM*) and chromatic dispersion (*D*) of the proposed wedge hybrid plasmonic THz waveguide as a function of the frequency from 0.1 to 2THz under optimized wedge tip angle of 100 deg and diameter of the Si nanowire cylinder of 40 μm. As shown in Fig. 4(a), the propagation length and figure of merit decreases first and then increases while the normalized mode area increases as increasing the frequency. The propagation length changes between 110.9 and 2698 mm, the normalized mode area increases from 3.45 × 10^−4^ to 2.02 × 10^−3^, and the figure of merit varies between 1.52 × 10^4^ and 4.88 × 10^4^ with the frequency changing from 0.1 to 2 THz. As shown in Fig. 4(c), one can see that the chromatic dispersion increases with the increase of frequency. An ultra-low chromatic dispersion varying from −1.3 × 10^−4^ to −12.3 ps/nm/km is achieved when changing the frequency from 0.1 to 2 THz.

### Fabrication method

According to the fabrication method used for hybrid wedge plasmonic waveguide at telecommunications frequency^23^, the focused-ion beam (FIB) technique could be used to form the metal wedge with high accuracy. After depositing a thin HDPE layer on the wedge metal as the low-index gap, a Si nanowire which could be prepared by vapor-liquid-solid method^27^ is placed on the HDPE layer above the metal wedge to form the hybrid wedge plasmonic waveguide. The Si nanowire is then covered by the HDPE cladding.

Alternatively, one could also fabricate the hybrid wedge plasmonic THz waveguide following a reverse step. The Si nanowire is first placed on a HDPE substrate and covered with HDPE cladding. The metal wedge will later be deposited after milling a V-shape groove in the upper HDPE layer, which is similar to the fabrication process for the wedge plasmon polariton waveguides^28^.

### Fabrication error tolerance

In view of the increased complexity of the proposed and designed wedge hybrid plasmonic waveguide, we also investigate possible practical fabrication issues (i.e. fabrication error tolerance).

**Figure 5:**
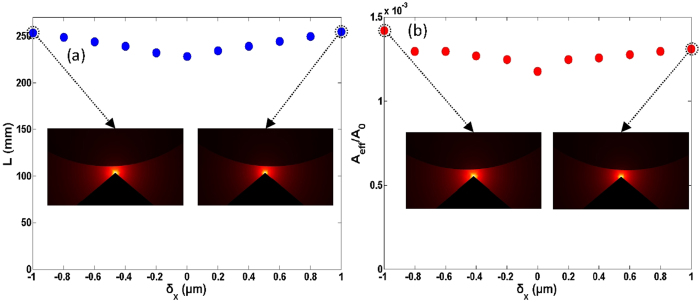
(**a**) Propagation length (*L*) and (**b**) normalized mode area (*A*_*eff*_ */A*_*0*_) vs. horizontal deviation of the Si nanowire from the metal wedge. The wedge tip angle is 100 deg, the diameter of Si nanowire cylinder is 40 μm, and the gap height is 1 μm.

It might be possible to control the size of the Si nanowire, the gap region, and the metal wedge with high precision^29^. However, accurate alignment between the Si nanowire and the metal wedge is not easy. We first study the impacts of the misalignment in the horizontal direction on the mode properties. Figure 5(a) show the propagation length and normalized mode area as a function of the horizontal deviation of the Si nanowire from the metal wedge. One can see less than 12% fluctuation of the propagation length and a small variation of the normalized mode area between 1.24 × 10^−3^ and 1.42 × 10^−3^ under ±5 μm misalignment in the horizontal direction.

**Figure 6:**
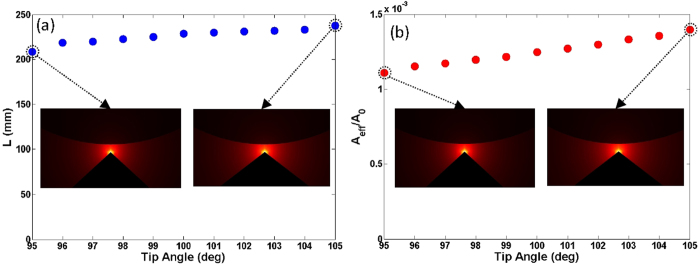
(**a**) Propagation length (*L*) and (**b**) normalized mode area (*A*_*eff*_ */A*_*0*_) vs. wedge tip angles. The diameter of Si nanowire cylinder is 40 μm, and the gap height is 1 μm.

Considering that the mode of the wedge hybrid plasmonic waveguide might be affected by the variation of the metal wedge tip angle, we then study the impacts of the variation of wedge tip angle on the mode properties. Figure 6(a) show the propagation length and normalized mode area as a function of the wedge tip angle varying from 95 deg to 105 deg. One can see less than 9% fluctuation of the propagation length and a small variation of the normalized mode area between 1.10 × 10^−3^ and 1.40 × 10^−3^ under ±5 deg offset from 100 deg.

**Figure 7:**
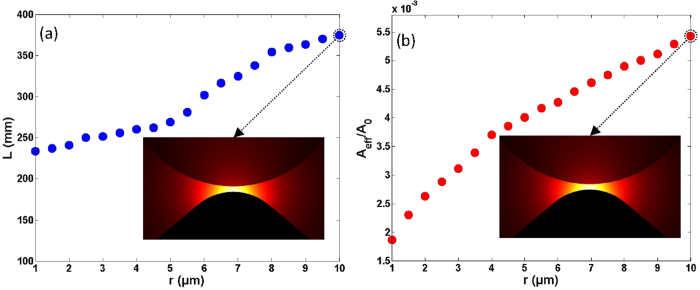
(**a**) Propagation length (*L*) and (**b**) normalized mode area (*A*_*eff*_ */A*_*0*_) vs. wedge tip curvature radius. The wedge tip angle is 100 deg, the diameter of Si nanowire cylinder is 40 μm, and the gap height is 1 μm.

Another possible practical fabrication issue is the wedge tip which might not be sharp but round. So we also study the impacts of the variation of wedge tip curvature radius on the mode properties. Figure 7(a) show the propagation length and normalized mode area as a function of the wedge tip curvature radius under a wedge tip angle of 100 deg. With a large increase of the wedge tip curvature radius from 1 to 10 μm, one can see the increase of the propagation length from 233 to 374 mm and the increase of the normalized mode area from 1.86 × 10^−3^ and 5.43 × 10^−3^.

The simulated results shown in Figs. 5, 6, 7 indicate that some of the fabrication errors in geometry parameters such as the misalignment between the Si nanowire and metal wedge in the horizontal direction, the variation of wedge tip angle, and the variation of wedge tip curvature radius have slight influences on the propagation length and normalized mode area. Consequently, the designed wedge hybrid plasmonic THz waveguide features favorable mode properties, i.e. long propagation length, ultra-small deep-subwavelength mode area, and large tolerance to the practical fabrication errors in geometry parameters.

## Discussions

In conclusion, we have proposed a wedge hybrid plasmonic THz waveguide which is formed by a Si nanowire cylinder above a triangular gold wedge with high-density polyethylene cladding. For given geometric parameters, the designed wedge hybrid plasmonic THz waveguide features long propagation length, ultra-small deep-subwavelength mode area, greatly improved figure of merit, and ultra-low chromatic dispersion. Additionally, the influences of possible practical fabrication issues on the mode properties have been analyzed in detail showing favorable tolerance to fabrication errors in geometric parameters.

The proposed wedge hybrid plasmonic THz waveguide takes the similar design to that at telecommunications frequency, which combines the advantages of SPP waveguide, slot waveguide, hybrid SPP waveguide and wedge SPP waveguide. So the fundamental physics of wedge hybrid plasmonic waveguide also originates from the synthesized operation mechanisms from SPP waveguide, slot waveguide, hybrid SPP waveguide, and wedge SPP waveguide^24^,^30^.

SPP is regarded as one kind of surface science at the interface between a dielectric and a metal. It is in essence light wave trapped on the interface between dielectric and metal as a result of the interaction between the light wave and free electrons of the metal. In-resonance surface collective oscillations of free electrons are excited by the electromagnetic fields of light wave. The resonant interaction between light wave and surface charge oscillations forms SPP. The electric field component of SPP is perpendicular to the surface, peaks at the interface, and decays exponentially away into dielectric and metal. The electric field in the vertical direction is evanescent with non-radiative nature which prevents power away from the surface. As a consequence, SPP waveguide can potentially break the diffraction limit. However, typical SPP waveguide with single metal-dielectric interface has no additional mode confinement in the dielectric side with more electric field penetration.

In addition to SPP, the discontinuity of electric field at the interface of two different dielectrics is known as another kind of surface science. The discontinuity of electric field is enhanced at the interface between high-contract-index dielectrics (one low-index dielectric and one high-index dielectric).

Slot waveguide is one example employing the surface science (two dielectric-dielectric interfaces). Typically, slot waveguide is formed by two high-contrast-index dielectric-dielectric interfaces. Slot waveguide guides light in the low-index slot region in which the electric field is greatly enhanced and tightly confined.

Hybrid SPP waveguide is also an example employing the surface science (one dielectric-dielectric interface and one dielectric-metal interface). Generally, a hybrid SPP or hybrid plasmonic waveguide is formed by one high-contrast-index dielectric-dielectric interface and one dielectric-metal interface guiding light in the low-index dielectric region. Two kinds of surface science, i.e. the discontinuity of electric field at the high-contrast-index dielectric-dielectric interface and the SPP at the dielectric-metal interface, contribute together to the supermode which is greatly enhanced in the small region of low-index dielectric. The supermode is also called hybrid mode which offers tight mode confinement with moderate propagation loss.

Wedge SPP is considered to be a kind of modified surface science with the surface shaped into a wedge. A wedge-shaped dielectric-metal interface forms a typical wedge SPP waveguide. By employing a wedge SPP waveguide and utilizing the extraordinary confinement property of the wedge plasmon polariton at the corner of the wedge, it is possible to achieve even smaller mode confinement.

The proposed wedge hybrid plasmonic THz waveguide borrows ideas from SPP waveguide, slot waveguide, hybrid SPP waveguide and wedge SPP waveguide and offers similar propagation length to hybrid SPP waveguide and similar mode confinement to wedge SPP waveguide. As a consequence, long propagation length and ultra-small mode area are both achieved in the designed wedge hybrid plasmonic waveguide. Remarkably, from SPP waveguide to slot waveguide, hybrid SPP waveguide, wedge SPP waveguide, and wedge hybrid plasmonic waveguide, the fundamental physics are all based on surface sciences, i.e. SPP at dielectric-metal interface, electric field discontinuity at high-contrast-index dielectric-dielectric interface, and wedge dielectric-metal interface. The superior mode properties of wedge hybrid plasmonic waveguide with long propagation length and ultra-small mode area benefit from respective distinct mode properties of SPP waveguide, slot waveguide, hybrid SPP waveguide, and wedge SPP waveguide.

Though the proposed wedge hybrid plasmonic THz waveguide features long propagation length and ultra-small mode area, the relatively increased complexity of the design raises the challenge of efficient input coupling, i.e. low efficiency of THz coupling could be another practical issue due to mode mismatch between the seed THz source and the primary mode of the THz waveguide. With future improvement, similar to the idea of enhanced coupling of THz radiation to cylindrical wire waveguides^31^,^32^, one might focus on the source of the THz radiation and design a special photoconductive THz source antenna. The specially designed geometry of the photoconductive antenna could radiate THz wave having spatially matched mode properties as guided in the wedge hybrid plasmonic THz waveguide. In this way it might be possible to greatly improve the coupling efficiency associated with the designed wedge hybrid plasmonic THz waveguide.

## Methods

The propagation length, defined as the distance from the input to where the mode field decays by a factor of 1/e^29^, is calculated by


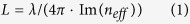


where Im(n_eff_) is the imaginary part of the effective mode index.

*A*_*0*_ is the diffraction-limited mode area and defined as λ^2^/4, A_eff_ is the ratio of the total mode energy to peak energy density written by


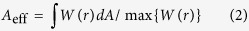


where W(r) is the energy density expressed as


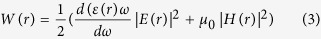


*FoM* is the ratio of the propagation length to the square root of effective mode area written by


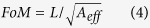


*FoM* is another important parameter providing an appropriate measurement for the trade-off between the propagation length and attenuation.

## Additional Information

**How to cite this article**: Gui, C. and Wang, J. Wedge hybrid plasmonic THz waveguide with long propagation length and ultra-small deep-subwavelength mode area. *Sci. Rep.* **5**, 11457; doi: 10.1038/srep11457 (2015).
